# Effect of 25‐Gauge Needle–Assisted Phacoemulsification of Dislocated Intravitreal Lens Nucleus

**DOI:** 10.1155/joph/8429779

**Published:** 2026-05-27

**Authors:** Qing Feng, Yuan Li, Yalin Jiang, Xincheng Sun

**Affiliations:** ^1^ Department of Ophthalmology, The Second People’s Hospital of Changzhou, The Third Affiliated Hospital of Nanjing Medical University, Changzhou, Jiangsu, China

**Keywords:** 25-gauge needle, complicated cataract surgery, dropped nucleus, pars plana vitrectomy, phacoemulsification

## Abstract

**Purpose:**

Purpose: To present and evaluate the effect of a novel technique, 25‐gauge (G) needle–assisted phacoemulsification, to remove dislocated intravitreal lens nuclei.

**Methods:**

Methods: Nine eyes of nine patients with dislocated intravitreal lens nuclei who underwent 23‐G pars plana vitrectomy (PPV) and 25‐G needle–assisted phacoemulsification between July 2022 and September 2024 were included in this study. Best‐corrected visual acuity (BCVA), intraocular pressure (IOP) and corneal endothelial cell count (CECC) were statistically analysed both preoperatively and postoperatively. The intraoperative and postoperative complications were evaluated. A minimum follow‐up of 6 months was completed for all eyes.

**Results:**

Results: All nine eyes of nine patients were successfully treated with 23‐G PPV and 25‐G needle–assisted phacoemulsification and postoperatively followed up for 6–13 months. BCVA improved significantly (*p* < 0.05) in all patients, and postoperative IOP was 16.0 ± 1.73 mmHg, remaining stable within the normal range. Although statistical differences were observed between preoperative and final follow‐up, the loss rate of CECC was within a tolerable range. No intraoperative and postoperative complications, such as iatrogenic retinal tears or vitreous haemorrhage, were observed.

**Conclusion:**

Conclusion: The 25‐G needle–assisted phacoemulsification is a safe and efficient alternative for removing dislocated intravitreal lens nuclei.

**Trial Registration:** ClinicalTrials.gov identifier: NCT06709378

## 1. Introduction

Dislocation of the lens nucleus into the vitreous cavity is a serious complication arising from complex cataract surgeries, ocular trauma or specific systemic conditions such as Marfan syndrome [1]. Several studies have reported its incidence in cataract surgery, ranging from 0.3% to 1.3%, depending on the type of cataract and the surgeon’s experience [2]. Residual lens nuclear material in the vitreous cavity can lead to various adverse reactions, including ocular inflammation, glaucoma, corneal oedema and cystoid macular oedema [3, 4]. Preserving eyesight necessitates the complete removal of dislocated nuclear material while minimising damage to the cornea, iris and retina. Pars plana vitrectomy (PPV) is currently the primary method for managing residual intravitreal lens fragments [5]. While small fragments and soft nuclei can be safely removed using a vitreous cutter, larger or hard nuclei pose challenges. Using the vitreous cutter alone proves less efficient and makes it difficult to fix the nucleus, leading to collisions with the retina, thereby increasing the risk of retinal damage [6]. Manipulating the dislocated lens nucleus into the vitreous cavity to a plane distant from the retinal surface prior to further removal procedures could minimise the rate of potential complications. Some materials and devices, such as perfluorocarbon liquids (PFCL) and frag bags designed for cystoscopic removal of ureteral and kidney stones, have been innovatively applied in the levitation of the posteriorly dropped lens away from the retina [1, 7].

We proposed a novel technique using a 25‐gauge (G) needle to assist phacoemulsification for the safe removal of dislocated intravitreal lens nuclei. Our technique requires no specialised instruments or materials to elevate the dislocated intravitreal lens to a safe plane for phacoemulsification. In this study, we reported this technique and evaluated its efficacy and safety.

## 2. Materials and Methods

Written informed consent was obtained from all patients. This study was approved by the institutional ethics committee (YLJSA169), adhered to the tenets of the Declaration of Helsinki and was registered at ClinicalTrials.gov (registration number: NCT06709378).

Between July 2022 and September 2024, nine patients presented with dislocated intravitreal lens nuclei due to various factors, including cataract surgery, trauma, connective tissue disorders and others, were included in this study. All patients underwent 23‐G PPV and 25‐G needle–assisted phacoemulsification of the dislocated intravitreal lens nucleus. All surgeries were performed by the same surgeon. Best‐corrected visual acuity (BCVA), intraocular pressure (IOP) and corneal endothelial cell count (CECC) were evaluated preoperatively and during postoperative follow‐up periods. BCVA, IOP, and CECC differences between the preoperative and final follow‐up were compared for statistical significance. Additionally, postoperative complications, such as iatrogenic retinal tears, retinal detachment and vitreous haemorrhage, were evaluated.

Data were analysed using SPSS Statistics 26.0 software (IBM Corp, Armonk, NY, USA). The BCVA was shown as a logarithm of the minimum angle of resolution (LogMAR) of visual acuity for descriptive and analytical purposes. The paired sample *t*‐test was conducted to analyse the preoperative and postoperative data of the parameters, including the BCVA, IOP and CECC. A *p* value of < 0.05 was considered to indicate statistical significance.

### 2.1. Surgical Technique

**FIGURE 1:**
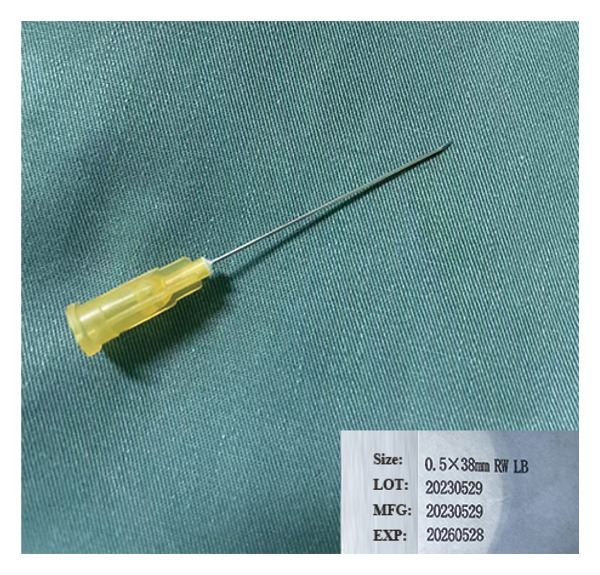
A 25‐gauge needle.

**FIGURE 2:**
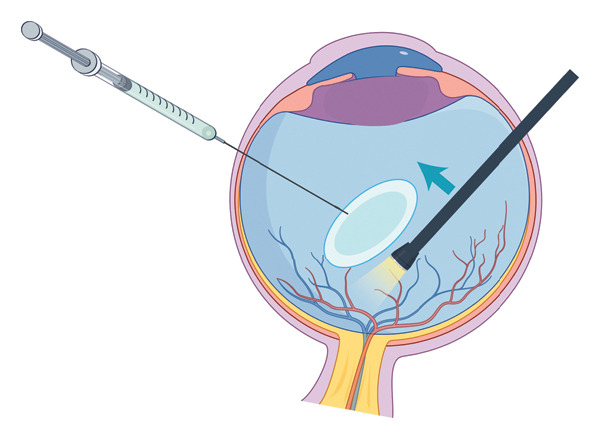
Graphical representation (by Figdraw).

**FIGURE 3:**
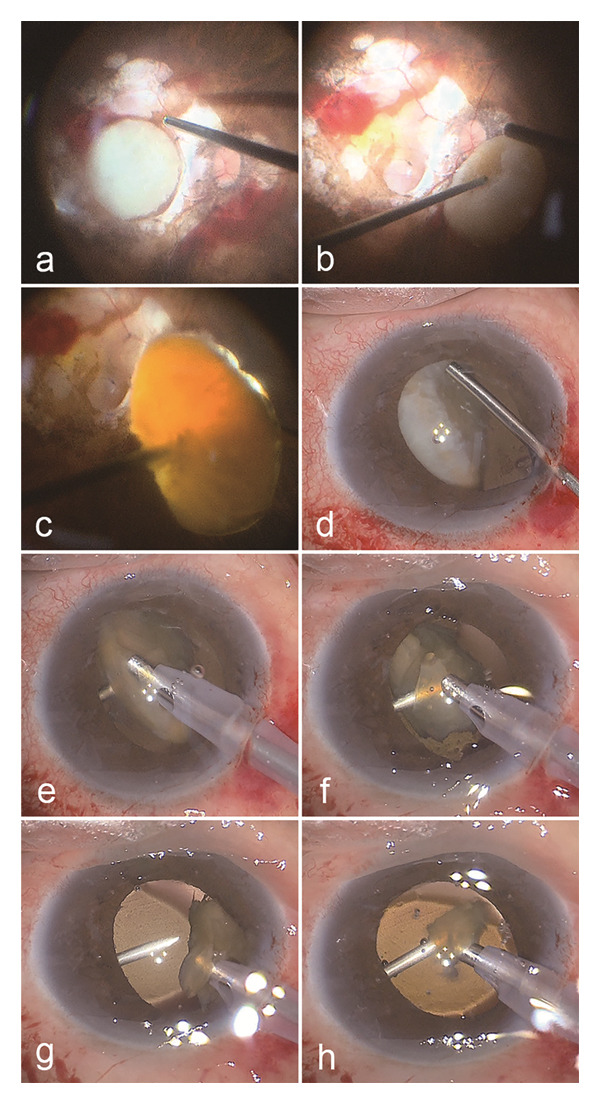
Surgical procedures. (a) The vitreous cutter was used to thoroughly remove adhesions between the lens nucleus and the retina. (b) The 25‐G needle tip pushed the nucleus outside the vascular arcade, after which the tip was gently inserted into the nucleus. (c) The nucleus was lifted by the needle to the centre of the vitreous cavity, and the light pipe helped in fully embedding the needle tip into the lens nucleus. (d) The nucleus was elevated to the iris plane. (e) The nucleus was rolled by the needle to enable uniform phacoemulsification. (f) As the phacoemulsification tip approached the needle wall or fissure caused by needle insertion, the probe maintained a high aspiration level throughout to securely suction the remaining lens nucleus. (g) Negative pressure was maintained to stabilise the nucleus, and the needle tip was slowly withdrawn from the nucleus. (h) The needle rotated and guided the nucleus into the probe until it was completely removed.

The operated eye underwent standard sterilisation procedures, followed by surface (oxybuprocaine 0.4%) and retrobulbar anaesthesia (lidocaine 2% and ropivacaine 0.2%) administration. For patients with dislocated intravitreal lens nucleus due to rupture of the posterior capsule during cataract surgery, the lens cortex and nucleus remaining in the capsular bag were first removed through a corneal incision using either the vitreous cutter or phacoemulsification probe. Subsequently, standard three‐port 23‐G PPV incisions were made. Triamcinolone acetonide was injected into the vitreous cavity to visualise the vitreous cortex. A complete vitrectomy was initially performed to release the adhesions around the dislocated lens nucleus and prevent retinal traction during subsequent procedures. A 25‐G needle (0.5 × 38 mm, shown in Figure 1) was gently inserted into the vitreous cavity through a scleral incision on the nondominant side. The needle tip lightly touched the nucleus, pushing it out of the retinal vascular arcade. The needle tip was gently inserted into the peripheral cortex of the lens nucleus, lifting the nucleus to the centre of the vitreous cavity, while the light pipe applied a pushing force from the opposite side to assist the needle tip in piercing the lens nucleus (shown in Figure 2). Key portions of this procedure were demonstrated in an accompanying video (see Additional file 1). Next, the nucleus was elevated to the iris plane. Then, a cataract phacoemulsification probe was inserted into the anterior chamber through a corneal limbal incision. The needle tip rotated the lens to ensure even emulsification of the nucleus, similar to the phaco‐rolling technique [8]. While cutting near the nuclear cleft created by the embedded needle tip in the hard nucleus, the phacoemulsification probe maintained a high aspiration level to stabilise the remaining lens nucleus and prevent it from falling into the vitreous cavity. Concurrently, the needle tip was slowly withdrawn from the nucleus, guiding the remaining nucleus into the probe until it was completely removed (see Additional file 2). If the entire lens nucleus with its capsule falls, the capsule can be removed using a vitreous cutter through the corneal limbal incision before the phacoemulsification of the nucleus. Finally, a thorough fundus examination was conducted to remove any remaining lens components and rule out retinal tears. Small fragments that fell into the vitreous cavity during phacoemulsification were removed using a vitreous cutter. If the dropped fragments are large, the aforementioned procedures can be repeated using the needle. The main surgical procedures of a typical case are shown in Figure 3. This typical case involved a patient with phacolytic glaucoma secondary to spontaneous lens dislocation into the vitreous cavity. A comprehensive ophthalmic examination revealed that this patient suffered from pathologic myopia, choroidal atrophy and subretinal haemorrhage. We successfully used this technique to emulsify the dislocated lens within a secure anatomical plane.

The choice of intraocular lens (IOLs) for fixation position and technique was determined based on the integrity of the zonules and capsular bag. For patients with no capsular bag or insufficient residual capsular bag to support the IOL, we chose to suspend IOLs via scleral fixation techniques. In patients with well‐preserved zonules and sufficient residual capsular bag to support the IOL, IOLs were placed in the ciliary sulcus.

## 3. Results

All participants presented with an intravitreal dislocated lens nucleus due to various reasons, including cataract surgery in five eyes, trauma in two eyes, spontaneous dislocation in one eye and Marfan syndrome in one eye. Each underwent phacoemulsification assisted by a 25‐G needle near the anterior chamber.

Clinical characteristics and baseline data are shown in Table 1. This study included four males and five females with a mean age of 76.9 ± 7.0 years (67–89 years). The Emery–Little classification for nuclear sclerosis was distributed in Grades 3–5. Seven patients underwent suspension of IOLs via scleral‐fixation techniques, and two patients were implanted with three‐piece IOLs secured in the ciliary sulcus. Postoperative follow‐up was 6–13 months. BCVA improved significantly at the final follow‐up postoperatively than preoperatively (0.50 ± 0.28 logMAR vs. 1.05 ± 0.17 logMAR, *p* < 0.01). The elevated IOP in Cases 2 and 3 preoperatively was due to secondary glaucoma attacks and was reduced to normal postoperatively. The postoperative IOP of the nine patients was 16.0 ± 1.73 mmHg. The mean CECC decreased from 2144.3 ± 218.5 cells/mm^2^ preoperatively to 2065.0 ± 217.9 cells/mm^2^ postoperatively. Although the difference was statistically significant, the CECC loss of 79.3 ± 69.4 cells/mm^2^ (approximately 3.7%) was within the tolerable range [9]. No related complications, such as bullous keratopathy, iatrogenic retinal tears, vitreous haemorrhage or retinal detachment, were noted in any of the patients during the postoperative follow‐up periods.

**TABLE 1:** Clinical characteristics of cases.

Case	Sex	Age	Eye	Emery–Little classification	Fixation method of IOL	Causes/time of lens dislocation	Dislocated material	BCVA pre/post (logMAR)	IOP pre/post (mmHg)	CECCs pre/post (cells/mm^2^)	Related complication	Follow‐up (months)
1	F	75	R	4	Scleral fixation	Cataract surgery/0D	whole nucleus	1.10/0.70	16/14	2334/2129	N	8
2	F	74	L	3	Scleral fixation	Traumatic/10Ds	3 quadrants	1.30/1.18	30/16	2498/2305	N	10
3	M	89	R	4	Scleral fixation	Cataract surgery/0D	3 quadrants	0.88/0.40	35/17	1942/1801	N	6
4	F	69	L	5	Via ciliary sulcus	Cataract surgery/0D	2 quadrants	1.18/0.30	19/13	2346/2089	N	12
5	M	78	R	4	Scleral fixation	Marfan syndrome/33Ds	whole lens	1.00/0.48	17/18	2245/1908	N	12
6	M	82	R	4	Scleral fixation	Spontaneous/7Ds	whole lens	0.80/0.40	15/17	1974/1878	N	13
7	M	67	L	4	Via ciliary sulcus	Cataract surgery/0D	whole nucleus	0.90/0.30	16/18	2042/1932	N	9
8	F	83	R	5	Scleral fixation	Cataract surgery/0D	2 quadrants	1.20/0.48	16/15	1857/1798	N	8
9	F	75	R	4	Scleral fixation	Traumatic/15Ds	whole lens	1.10/0.30	21/16	2061/1908	N	10

*Note:* F = female; R = right; L = left; IOL = intraocular lens; D = day; IOP = intraocular pressure; N = none.

Abbreviations: BCVA, best‐corrected visual acuity; CECC, corneal endothelial cell count.

## 4. Discussion

Various factors can lead to the dislocation of the lens into the vitreous cavity. Common causes include posterior capsule rupture during cataract surgery and traumatic or spontaneous lens dislocation. Lens nucleus dislocation resulting from posterior capsule rupture during phacoemulsification is relatively common, particularly among less experienced ophthalmic surgeons and trainees [10]. Studies indicate that the rate of nucleus drop in phacoemulsification procedures performed by trainees can reach 1.1% [11]. Residual lens fragments, particularly nuclear material left in the vitreous cavity, increase the risk of complications, including corneal oedema, uveitis, cystoid macular oedema, retinal detachment and glaucoma [3, 4]. These complications significantly impact the patient’s visual prognosis, and timely removal of the dropped lens is crucial. Therefore, developing a safe and easily mastered technique to manage the dropped lens nucleus into the vitreous cavity is significantly important for enhancing surgeons’ ability to deal with such complications and reducing risks associated with secondary surgery.

The management of dislocated lenses varies depending on their composition. Generally, small and less dense lens fragments (Emery–Little Grades 1–2), particularly those containing minimal nuclear material, may be removed via a three‐port PPV using a vitreous cutter alone at a reduced cutting speed, as these nuclei are more easily fragmented and aspirated without the need for additional stabilising techniques. [12]. However, managing larger and denser nuclei (Emery–Little Grades 3–5) is challenging with the vitreous cutter alone due to the limitations imposed by the cutter’s diameter and cutting speed, particularly as vitreous surgical equipment has evolved from 20G to 23G, 25G, and even finer 27G, making such procedures more complex and time‐consuming. Moreover, fixing the lens or its fragments is challenging during PPV, as nucleus fragments repeatedly fall onto the retina and collide with it, increasing the risk of retinal damage [12]. Berry et al. reported a method of using the 1.5 French N‐Circle Nitinol Tipless Stone Extractor (Cook Medical, Bloomington, IN), originally designed for cystoscopic removal of ureteral and kidney stones, to fix the dislocated lens nucleus in the mid‐vitreous cavity for efficient removal with a vitreous cutter. Limitations of this reported technique include the requirement for an extra chandelier endoilluminator, bimanual dexterity and an additional assistant. In addition, the force used to tighten the extractor is difficult to control with precision, and too much force will fracture the nucleus into multiple small pieces, complexing the surgical procedures [1].

Röver first reported the dislocated nucleus being emulsified within the vitreous cavity using a phacoemulsification probe. The frequency and energy of intravitreal ultrasound need to be tightly controlled. Otherwise, this technique may increase the risk of retinal tears and detachment [13]. Jimenez et al. proposed a technique using microinterventional nucleus disassembly to reduce the energy required for fragmatome lensectomy. This technique used the microinterventional microfilament loop device (miLOOP) (Zeiss, Oberkochen, Germany) to facilitate intravitreal dislocated lens disassembly and reduce phacoemulsification energy in the vitreous cavity, decreasing the risk of retinal damage but requiring a larger scleral incision [14].

Although improved vitrectomy instruments avoid larger incisions, the high efficiency of removing the dropped nucleus was significantly limited, especially for hard nuclei, due to the inherent fluidic limitations of the vitreous cutter’s lumen [15]. Based on the concepts of retinal protection and ensuring surgical efficiency, Jang proposed using PFCL to float the dislocated lens nucleus from the bottom of the vitreous cavity to the iris plane [7], and phacoemulsification of the lens can be safely performed away from the retina and cornea. Nevertheless, the curved tension surface of PFCL disperses small lens fragments toward the periphery and behind the iris, rendering them difficult to visualise and complicating their removal [16]. Complete removal of all PFCL from the vitreous cavity is imperative due to the retinal toxicity associated with residual PFCL [17, 18]. However, owing to the effects of phacoemulsification procedures and the difficulty in thoroughly removing peripheral vitreous, PFCL readily forms dispersed fine droplets that are challenging to eliminate completely, thereby increasing the complexity of the surgical procedures.

The methods and techniques reported for managing the intravitreal dislocated nucleus typically require specialised instruments and auxiliary materials. Based on the principle of performing phacoemulsification at a safe distance from the retina and cornea, we developed the novel technique that requires no specialised materials or instruments. Our technique only requires a readily available 25‐G needle, using the friction between the needle tip and the lens cortex to facilitate the elevation of the nucleus to the iris plane for phacoemulsification. Notably, the crystalline lens increases in weight with age, reaching approximately 255 mg in adulthood [19]. Therefore, even a small amount of friction between the needle tip and the lens cortex may be sufficient to achieve effective lifting of the dislocated nucleus. In contrast to previously reported approaches such as the ‘kebab technique’, which employs a bipolar pencil for nucleus penetration and stabilisation [6], the present method avoids the use of intraocular thermal energy and relies solely on mechanical interaction. The sharp tip and adequate mechanical rigidity of the needle enable controlled penetration and stable engagement of the dislocated nucleus, which may reduce the risk of thermal injury to adjacent retinal tissues while simplifying intraoperative handling. The concise procedural workflow allows positioning of the operative plane away from the retina and cornea, potentially reducing surgical time and the risk of intraoperative and postoperative complications. These features also make the technique relatively intuitive and facilitate its adoption by vitreoretinal surgeons. Several key points of needle‐assisted phacoemulsification of the dislocated lens are as follows: First, to prevent retinal damage in the macula, it is essential to push the lens outside the vascular arcade for safe needle insertion; second, to avoid stretching the retina and reduce resistance when lifting the lens, ensure that adherent vitreous fibres around the dislocated lens nucleus are completely removed before inserting the needle into the vitreous cavity; third, as the phacoemulsification tip nears the needle wall or fissure caused by the insertion of the needle, the probe maintains a high aspiration level throughout to fix the remaining lens nucleus and prevent it from dropping; and fourth, if dropped fragments exceed 2 mm in diameter during phacoemulsification and cannot be removed easily by a vitreous cutter , the aforementioned procedure can be repeated using the needle.

With the increased rate of cataract surgery, patients with rock‐hard nucleus cataracts have become relatively rare in clinical practice [20]. No patients with rock‐hard nucleus cataracts without a cortical cushion were included in this study. For this type of nuclei, lacking the lens cortical cushion [21], the insertion of the needle tip into the lens may require a large force, which could easily damage the retina, while insertion too superficially cannot provide enough friction to lift the hard nucleus. The efficacy of removing such nuclei requires further verification. Additionally, further prospective studies with larger sample sizes and comparative control groups, together with extended follow‐up periods, are required to more comprehensively validate the safety and long‐term efficacy of this technique and to enhance the generalisability of the findings.

In conclusion, our study demonstrates that 25‐G needle–assisted removal of the dislocated intravitreal lens nucleus is an effective and safe technique. This technique has a wide range of applications and decreases the risk of related complications.

## Funding

The authors declare that no funds, grants, or other support were received during the preparation of this manuscript.

## Conflicts of Interest

The authors declare no conflicts of interest.

## Supporting Information

Additional supporting information can be found online in the Supporting Information section.

## Supporting information


**Supporting Information 1** Additional file 1: The dislocated intravitreal lens was elevated to the vitreous cavity using a 25‐G needle.

**Supporting Information 2** Additional file 2: The phacoemulsification of the lens nucleus was performed near the anterior chamber.

## Data Availability

The data that support the findings of this study are available from the corresponding author upon reasonable request.
